# On Spontaneous Dispersion as a Cause of Microstratification of Metal Melts

**DOI:** 10.3390/ma17102215

**Published:** 2024-05-08

**Authors:** Olga A. Chikova, Vladimir S. Tsepelev, Kseniya Yu. Shmakova

**Affiliations:** 1 Departament of Physics, Ural Federal University, Yekaterinburg 620002, Russia; o.a.chikova@urfu.ru; 2 Research Center for Physics of Metal Liquid, Ural Federal University, Yekaterinburg 620002, Russia; k.y.shmakova@urfu.ru

**Keywords:** liquid alloys, microstratification, dispersed particles, spontaneous dispersion, criteria for true (entropic) spontaneous dispersion, mechanisms of quasi-spontaneous dispersion, methods and results for assessing size of dispersed particles

## Abstract

The phenomenon of spontaneous dispersion is considered as the cause of the microstratification of metal melts. In a microstratification melt, a violation of long-range order in the arrangement of atoms (LRO) is observed, which corresponds to a dispersed particle size of more than 2 nm. Microseparation occurs due to spontaneous dispersion upon contact of liquid and solid metal or the mixing of two liquid metals. The possibility of spontaneous dispersion was assessed using three different criteria: Volmer’s cr iterion, Rehbinder’s criterion and the diffusion rate criterion. The diffusion rate criterion was obtained on the basis of the theory of rate processes, which describes how diffusing atoms overcome the interphase boundary. It has been established that Al–Sn melts contain colloidal-scale particles (4 nm), and Al–Si and Al–Ge melts contain atomic-scale particles (0.1 nm). For a system with a continuous series of Cu–Ni solid solutions in dispersion (Cu10Ni90—Cu20Ni80), the particle size is 2 nm. The particle size of the ternary eutectic GaInSn in the dispersion (Ga50In50—Ga50Sn50) is 5.6 nm, and the size of immiscible Cu–Fe melts in the dispersion (Cu80Fe20—Cu60Fe40) is 4.8 nm. Long-range order violations (LRO) and the presence of microlayering with colloidal particles larger than 20 nm were observed in the GaInSn ternary eutectic, in the Al–Sn simple eutectic with the preferential interaction of similar atoms, and in Cu–Fe melts with a monotectic phase diagram.

## 1. Introduction

The development of ideas about microinhomogeneities in metal melts from a scientific point of view is relevant to the understanding of the physicochemical nature of temperature-induced liquid–liquid transitions (TI-LLST), which are associated with the irreversible destruction of microinhomogeneities when the melt is heated to a temperature T*. TI-LLST is characteristic of multicomponent metal melts and plays an important role in the final microstructure and properties of alloys. By measuring the temperature dependence of the viscosity, density, electrical resistivity and surface tension of the melt, the temperature T* can be experimentally determined and explained by a structural transition caused by the destruction of microinhomogeneities [1]. The TI-LLST model is based on the concept of “heterogeneous liquid—homogeneous liquid” and is interpreted as a structural transition from a heterogeneous system to a homogeneous solution when the melt is heated to the temperature T*. The idea of the model is to theoretically determine the temperature T* at which the viscosity of a heterogeneous system becomes equal to the viscosity of a homogeneous liquid solution with a uniform distribution of atoms. It is proposed to calculate the viscosity of a heterogeneous melt using the expression for heterogeneous media based on the unit cell method for a geometric model of isolated inclusions, and the viscosity of a homogeneous melt using an additive dependence. The model demonstrates the possibility of percolation phenomena occurring in heterogeneous melts, and determines the limiting ratio of the viscosity of the medium and the inclusion at which a percolation transition is possible [2].

The classification of TI-LLST is based on the scale of micro-inhomogeneities found in liquid alloys. Microinhomogeneities can occur due to the preferential interaction of identical or different atoms, which can result in a violation of short-range order (SRO) in the atomic arrangement. These microinhomogeneities typically have a size of 0.2–0.5 nm. The microinhomogeneous state of melts, caused by the segregation of atoms of a fluctuation nature without clear interphase boundaries (clusters), is associated with a violation of mean order (MRO); clusters typically have a size of 0.5–2 nm. Melt microstratification corresponds to a violation of long-range order (LRO) in the atomic arrangement. The phenomenon of microseparation refers to the presence of dispersed particles that are enriched in one component and suspended in a medium of a different composition, resulting in a clear interphase surface. It is worth noting that colloidal particles are typically larger than 2 nm [1]. The present work aims to explore the microstratification of melt that occurs as a result of the spontaneous dispersion of solid metals in liquid metals or the spontaneous dispersion and mixing of two liquid metals.

Concepts concerning the microlayered (colloidal) structure of liquid alloys related to eutectic and monotectic systems are consistently developed by P.S. Popel. [3]. Based on small-angle neutron scattering experiments in melts of Pb–Sn and Al–Si eutectics, P.S. Popel, U. Dahlborg and M. Calvo-Dahlborg experimentally substantiated the concept of the microstratified (microheterogeneous) state of liquid multicomponent alloys. Regions enriched in one of the elements and separated from the rest of the liquid alloy by a transition layer have been detected. Two families of particles have been identified: small particles with a size of 1–4 nm and large particles with a size of up to 9 nm; it has been shown that with increasing temperature, the particles dissolve and recombine into smaller ones [4].

The concept of eutectic melts having a colloidal structure was first proposed by Yu. A. Klyachko [5], and later developed by A.A. Vertman, A.M. Samarin, and their colleagues [6]. They viewed eutectic melts as classical colloidal systems with particle dispersion ranging from 1 to 10 nm. From a physical chemistry perspective, the melt is a microheterogeneous system, also referred to as “microstratified” or in a “colloidal state”. V.M. Zalkin proposed that eutectic alloys in the liquid state form thermodynamically stable two-phase microemulsions. These microemulsions are created due to the delayed dissolution of one of the components and gradually transition into a homogeneous solution. The microemulsions are lyophilic two-phase systems [7]. The transition from microemulsion to homogeneous solution is reversible; upon cooling, the original microheterogeneity is restored.

A stable colloidal system was questioned by A.A. Vertman due to the violation of the phase rule at the eutectic point [6]. However, it has been pointed out by Frenkel [8] that this statement may be inconsistent, as an additional degree of freedom appears when one of the phases is dispersed to colloidal scales, due to the pressure inside the dispersed particles or their radius [9]. The assessment of dispersed particle sizes in liquid alloys, particularly the microheterogeneous (colloidal) structure, has been a topic of interest. Physical experiment data have been used to determine the sizes of these particles. Various experiments such as the centrifugation of liquid cast iron (A.A. Vertman, A.M. Samarin and A.M. Yakobson), sedimentation experiments for melts in the Al–Si system (I.V. Gavrilin), electron diffraction, ultraacoustic experiments, and studies of small-angle X-ray scattering have been conducted. These experiments have shown that the dispersed particle size is approximately 10 nm [3].

The viscosity–temperature relationship of microheterogeneous metal melts was analyzed using the theory of rate processes [10]. This analysis provided a numerical estimate of the size of dispersed particles in metal melts of components that interact eutectically and monotectically, which was found to be 1–5 nm [10]. Using equations proposed by G. Kaptay [11] for a regular solution in the Gibbs formalism, it was found that dispersed Fe–C particles in a Mn–C environment could range in size from 2 to 34 nm and still maintain thermodynamic stability [12]. Please note that at a T = 1900 K for the Fe-10%Mn-0.9%C melt with a dispersed particle radius r > 7 nm, the excess free energy of the transition layer at the particle–medium boundary has a negative value, which, according to [11], is a condition for the spontaneous dispersion of the system, i.e., dispersed particles with a size consistent with data on the size of structural units of viscous flow obtained earlier within the framework of the theory of rate processes [13].

One of the stages in the evolution of a particle of a charge material dissolving in a metal liquid may be its spontaneous dispersion. The theory of the spontaneous dispersion of solid metals upon contact with a metal melt in eutectic systems is considered in detail [14]. The conditions for the spontaneous dispersion of coal were studied by the authors of [15], who believed that this is possible if the value of the specific free surface energy σ_m_ of the interface between solid and medium satisfies the Rebinder criterion [16]. This can be achieved by taking the size of coal particles in dispersion equal to the maximum size of particles that can participate in Brownian motion, which is δ = 5 × 10^−6^ m. It was found that σ_m_~10^−9^ J/m^2^. Thus, to achieve the true spontaneous dispersion of solids in a liquid, it is necessary to reduce the interfacial free energy to 10^−1^ J/m^2^.

The phenomenon of spontaneous dispersion can be divided into two distinct phenomena. The first is the true spontaneous dispersion of particles of colloidal sizes, which leads to the formation of thermodynamically stable lyophilic systems that are prone to collective recrystallization. The second is the formation of coarse dispersions [15]. The thermodynamic condition for true spontaneous dispersion (entropic) is a decrease in the system’s free energy, which compensates for the increase in free surface energy during the formation of particles with a well-developed surface. This occurs due to an increase in the system’s entropy resulting from the involvement of generated particles in Brownian motion. Quasi-spontaneous dispersion occurs when solid metals come into contact with liquid ones, which is known as liquid metal embrittlement (LME). There are three mechanisms of LME: the Rehbinder effect, the Lynch model, and the Robertson model.

The article is devoted to the theoretical study of the patterns of spontaneous dispersion when mixing two liquid metals as the cause of the micro-stratification of the resulting melt. The aim is to estimate the sizes of dispersed particles in microlayered melts for systems with different types of phase diagrams.

## 2. Calculation Methodology

- The laws of true (entropy) spontaneous dispersion

The conditions for the spontaneous dispersion of solid metals in contact with a metal melt were studied by P.A. Rebinder, E.D. Shchukin and A.V. Pertsov [17]. Shchukin noted that the dispersion of the macrophase is thermodynamically favorable if the change in free energy due to dispersion (the release of n particles of radius r, at a sufficiently low interphase energy σ) is negative, i.e., $∆F=n4\pi \sigma r^{2}-T∆S<0$, where ∆S(C) is the increase in entropy, and C is the concentration. In the presence of a factor that prevents process dispersion in the limit molecular sizes b, a negative minimum ∆F may arise at r < b, i.e., a thermodynamically stable colloidal system is formed. The analysis of the behavior of function ∆F = ∆F(r,σ,n,C) for three different conditions gives us:

(i) Constant C with a virtual maximum;
(ii) r is a constant with a negative minimum;
(iii) n is constant when this function is monotonic in all cases for monodisperse systems with a wide variation in σ.

In all three cases, the equation ∆F = 0 is a necessary condition for spontaneous dispersion and the formation of a thermodynamically stable lyophilic colloidal system. At normal temperatures and low concentrations, this requires small-sized particles of around 10 nm and a minimum σ of 10^−2^–10^−1^ mJ/m^2^. These conditions become “simpler” for the dispersion of aggregates (for example, σ is on the order of unity) and “more complex” for highly concentrated systems (in this case, σ decreases to $10^{-3}$ mJ/m^2^) [18].

The first thermodynamic analysis of stability of lyophilic colloidal systems was made by M. Volmer [19]. Volmer studied the formation of lyophilic emulsions at temperatures slightly below the temperature of absolute mixing of two liquids, i.e., in the critical region. The main achievement of Vollmer’s work was obtaining an expression to estimate the interfacial tension at which the formation of lyophilic colloidal systems can be observed. Volmer determined the average volume of colloidal particles using the following expression:

(1) $$<V>=\left[\int_{0}^{\infty }\frac{4}{3}\pi r^{5}exp(-\frac{4\pi r^{2}\sigma }{kT}dr\right]/\left[r^{2}exp(-\frac{4\pi r^{2}\sigma }{kT}dr\right]$$

Volmer obtained the following relationship by integrating expression (1) and expanding exponential factors in a series in powers of r^2^: the relationship between the average volume up to which the spontaneous dispersion of particles is beneficial,$\;\bar{V}$, and the value of interfacial tension at their boundaries σ:

(2) $$\bar{V}=\frac{4}{\sqrt{\pi }}(\frac{4\pi \sigma }{kT}{)}^{\frac{-3}{2}}$$

Therefore, r=0.27kTσ m, and for a high-temperature colloidal system (T = 1000 K and r = 10 nm), σ should be below 10^−5^ J/m^2^. Volmer was able to obtain an expression for estimating interfacial tension $\sigma_{1}$, at which the formation of lyophilic colloidal systems can be observed in the form:

(3) $$\sigma_{1}=\frac{0.08kT}{r^{2}}$$

The formation of a lyophilic colloidal system can occur when the increase in surface free energy during the formation of a colloidal particle (~σr^2^) is close to the energy of thermal motion (~kT). In this case, the thermodynamic advantage of the dispersion process is associated with an increase in the entropy of the system with the formation of a large number of colloidal particles. At low σ, the increase in entropy compensates for the increase in free energy associated with an increase in interfacial area. Rebinder [20] obtained the following expression for the limit value of surface tension σ_2,_ at which spontaneous dispersion becomes thermodynamically favorable:

(4) $$\sigma_{2}=\frac{(10-15)kT}{r^{2}}$$

If r = 10 nm is assumed, then σ_2_~10^−3^ J/m^2^, which qualitatively confirms the result obtained by Volmer (3). Thus, the feasibility of spontaneous dispersion depends on the ratio of the sizes of the dispersed particles and the magnitude of the interfacial tension at their boundaries.

The criterion for spontaneous dispersion can be obtained on the basis of the ideas “Theory of rate processes: kinetics of chemical reactions, viscosity, diffusion and electrochemical phenomena” [21] related to overcoming the interphase boundary by diffusion atoms. In this case, the potential energy profile along the diffusion path is given by a periodic function with period δ. If the concentration of atoms in 1 cm^2^ in two layers separated by a distance δ is equal to c_1_ = c and c_2_ = c + δdc/dx, then the number of atoms moving from left to right through the potential barrier is equal to J_1_ = N_A_c_1_δk (particles/cm^2^), the flow in the opposite direction is equal to J_2_ = N_A_c_2_δk and the resulting flow is equal, and according to [21]:

(5) $$J=J_{2}-J_{1}=N_{A}\delta k(c_{2}-c_{1})=N_{A}\delta^{2}k\frac{dc}{dx}=D\frac{dc}{dx}$$

Rate constant k (i.e., the number of transitions of atoms from one equilibrium position to another in 1 s):

(6) $$k=(\frac{kT}{h})\cdot \left(\frac{F^{++}}{F}\right){exp}^{\varepsilon_{0}/kT}$$

where F^++^ and F are sums over states in the activated position and the equilibrium position,

(7) $$F/F^{++}=(2\pi mkT{)}^{1/2}\cdot {\upsilon_{f}}^{1/3}$$

$\upsilon_{f}$ is the fluctuation free volume, and N_A_ is the Avogadro number.

Let’s consider the process of dissolution of a substance, i.e., diffusion through the interfacial surface. The flow of the substance in the direction of dissolution is expressed as follows:

(8) $$J=J_{2}-J_{1}=N_{A}\delta kc_{1}(\exp(-\frac{\Delta F}{2kT})-\exp(\frac{∆F}{2kT}))-N_{A}\delta^{2}k\frac{dc}{dx}\exp(\frac{\Delta F}{2kT})\approx N_{A}kc_{1}\delta \frac{\Delta F}{kT}-{-N}_{A}\delta^{2}k(\frac{dc}{dx}+\frac{\Delta F}{2kT})\approx N_{A}kc_{1}\delta \frac{\Delta F}{kT}-N_{A}\delta^{2}k\frac{dc_{1}}{dx}$$

where ΔF is the change in free energy due to the presence of the interfacial surface. The first term in expression (8) “controls” the transition of atoms across the interphase surface and competes with the second term. In fact, ΔF = r^2^ σ, where σ is the surface tension coefficient. If there is no flow of atoms across the interface, then

(9) $$N_{A}kc_{1}\delta \frac{\Delta F}{kT}=N_{A}\delta^{2}k\frac{dc}{dx}$$

From here,

(10) $$c_{2}=c_{1}\exp(\sigma r^{2}/2kT)$$

Since $\sigma r^{2}<<2kT$, the exponential function in expression (10) can be expanded into a series and limited to the first terms. From here we get:

(11) $$c_{2}=c_{1}(1+\sigma r^{2}/2kT)\;\mathrm{or}\;\frac{c_{2}-c_{1}}{c_{1}}=\sigma r^{2}/2kT$$

The critical value of surface tension at which spontaneous dispersion becomes thermodynamically favorable can be determined from the condition $c_{2}\leq c_{1}$. Therefore,

(12) $$r<1.4\sqrt{kT/\sigma }$$

Thus, the authors obtained the diffusion rate criterion for the critical value of interfacial tension for spontaneous dispersion at a given temperature:

(13) $$\sigma_{3}=\frac{2kT}{r^{2}}$$

This result (13), as well as expression (12) obtained above, are both consistent with the ideas of M. Volmer and P.A. Rebinder [17].

**Figure 1:**
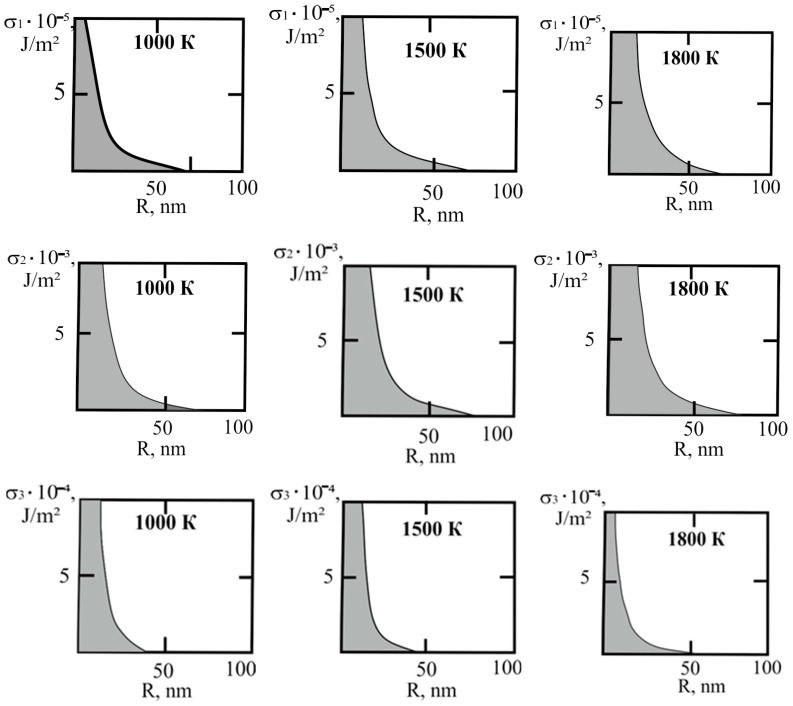
Criteria for spontaneous dispersion: σ_1_—according to Volmer; σ_2_—according to Rehbinder; σ_3_—according to the diffusion rate criterion estimates [14]. The shaded area corresponds to the lyophilic system.

Figure 1 shows the dependence of the critical value of interfacial tension on the radius of a dispersed particle at temperatures of 1000 K, 1500 K, and 1800 K, according to three different criteria for spontaneous dispersion: M. Volmer (3), P.A. Rebinder (4), and the diffusion rate criterion [14] (13). 

“Theory of rate processes: kinetics of chemical reactions, viscosity, diffusion and electrochemical phenomena” [21] can be used to estimate the critical interfacial tension at the boundary between the dispersed particle and dispersion medium. To do this, the kinetic unit of viscous flow is taken as a dispersed particle, and its size can be estimated from the results of a real viscometric experiment (Table 1). According to theory [21], the fluctuation free volume for a cubic packing of particles is equal to

(14) $${\upsilon_{f}}^{1/3}=-(2RT\upsilon^{1/3})/\Delta {Ε}_{evap}$$

According to Stefan’s rule [22], the surface tension of a liquid is:

(15) $$\sigma =\Delta E_{evap}N_{A}^{-1/3}V_{\mu }^{-2/3}$$

From expressions (14) and (15), it follows that

(16) $${\upsilon_{f}}^{1/3}=(2RT\upsilon^{-1/3})/(N_{A}\sigma )$$

As in the theory [21], we will assume that the sizes of the vacancy and the particle are equal; then, $\upsilon_{f}=8\cdot (2d-d{)}^{3}=8d^{3}$ and $\upsilon =8d^{3}$, and expression (15) can be written in the form:

(17) $$\sigma_{3}=kT/2d^{2}=2kT/r^{2}$$

which corresponds to Equation (13) and to the result obtained earlier by Summ [22,23,24]. B.D. Summ established a connection between surface tension and the heat of fusion of metals, and concluded that surface tension occurs due to a phase transition in the form of the solidification of the surface layer (SL). The physical state of the SL liquid corresponds to a solid phase. At the melting temperature, this thin layer, which is up to several atomic diameters, is continuous. At higher temperatures, the solid phase forms a «network structure». As the temperature increases, the proportion of solid phase in the SL decreases [24]. The SL grid model makes it possible to explain the decrease in the surface tension of the melt with increasing temperature and the change in interfacial tension at the «dispersed particle–melt» boundary in microinhomogeneous melts. Table 1 presents the interfacial tension results at the “dispersed particle–melt” boundary, calculated using Formula (12) and the SL grid model. The microinhomogeneities’ characteristic size, d (nm), was determined based on the results of viscometry theory [21], which approximates the temperature dependences of the melt viscosity with equations of the form [10]:

(18) ν(T)=Bd−12T12eε0kT

The size dependence of interfacial tension at the boundary of structural units of viscous flow (17) obtained is consistent with the previously obtained criterion for spontaneous dispersion (13). Assessing the interfacial tension of structural units of viscous flow (refer to Table 1) at T = 970 K using Formula (17) indicates consistency in magnitude with the previously calculated interfacial tension values obtained from analyzing experimental data under the assumption of complete wetting:

(19) $$\sigma^{\left(1-2\right)}=\sigma^{\left(1\right)}-\sigma^{\left(2\right)}$$

for particles 4–6 nm in size. Larger particles introduced into the melt during the melting of the phase components of a heterogeneous sample will be destroyed by spontaneous dispersion to a given size. A viscometric study can indicate whether thermodynamic equilibrium has been established between the dispersed particles and the environment in a microheterogeneous melt. A method is shown for estimating the surface tension value at the inclusion boundary during the dissolution of charge material fragments, and also predicts the degree of spontaneous dispersion during melt formation [14].

It has been established that Al–Sn melts have colloidal-scale particles, while eutectic melts with a predominant interaction of different types of atoms, such as Al–Si and Al–Ge, have atomic-level particles (Table 1). Therefore, a viscometric study of melts can help predict the conditions for spontaneous dispersion during the alloy formation process and judge the change in interfacial tension at the “dispersed particle–melt” boundary [14].

G. Kaptai’s approach offers a new way to assess the possibility of true spontaneous dispersion in a microheterogeneous melt. It is important to note that a negative excess free energy of the transition layer at the boundary of the dispersed particle and the medium is a condition for spontaneous dispersion, according to [11]. The negative surface tension condition of a binary regular solution is discussed using the recently validated Butler equation [25]. It has been demonstrated that surface tension becomes negative only for solutions with strong repulsion between atoms of different components. This repulsion must be so strong that the phenomenon occurs only inside the mixing zone, which is the two-phase region of macroscopic liquid solutions. Negative surface tension is only possible in a nonequilibrium state for macroscopic solutions. However, it has been demonstrated that nanoemulsions and microemulsions can be thermodynamically stable, preventing coalescence and phase separation. A thermodynamic theory of emulsion stability is developed for a three-component (A-B-C) system. A-rich droplets are dispersed in a C-rich matrix, separated by a segregated B-rich layer. The solubility of B is limited in both A and C, and the mutual solubility of A and C is neglected. The theory shows that when a critical droplet size is reached by forced emulsification, it is replaced by spontaneous emulsification. Subsequently, the droplet size decreases to an equilibrium value. The existence of a maximum temperature of emulsion stability is shown. In low-energy emulsification, spontaneous emulsification can occur below this maximum temperature, increasing as the temperature decreases further. This discovery can be applied to interpret experimental observations of spontaneous emulsification or to develop stable microemulsions and nanoemulsions [11].

- **Regularities of quasi-spontaneous dispersion**

G.M. Bartenev [14] found a less strict condition for spontaneous dispersion compared to condition (2). Bartenev analyzed crack development in the presence of an adsorption-active medium using the fluctuation theory of destruction of solids. According to Bartenev, the thermodynamic condition for spontaneous dispersion occurs when the safe stress in the medium is equal to the additional “breaking” stress created by the medium. The dispersion process can occur when the solid body’s specific free surface energy decreases by about one order of magnitude, approximately 10^−2^–10^−1^ J/m^2^. The medium’s “disjoining” effect is defined as $\left(\sigma_{0}-\sigma \right)$, where σ_0_ is the surface tension of a solid in a vacuum, and σ is in the medium. According to A.V. Pertsov [14], Bartenev’s theory of spontaneous dispersion can be applied to the phenomenon of distribution of adsorption-active liquid metals along the grain boundaries of polycrystalline metals.

In [26], a study was conducted on the spontaneous dispersion of solids in a medium with surfactants, known as quasi-spontaneous dispersion. A surface-active medium, close in molecular nature to a solid, reduces its surface tension, and brittle fracture is observed even at low tensile stresses. The surface energy of a solid can be reduced to the point where the colloidal state becomes more thermodynamically stable, causing the body to spontaneously disintegrate into parts without external stress. The kinetics of this type of destruction are determined by the presence of structurally weakened boundaries between parts and internal stresses of the second kind at these boundaries. A rupture cannot occur solely due to a decrease in surface tension; the presence of tensile stresses at the crack tip is necessary. As stated in [26], spontaneous dispersion of solids can occur if the total tensile stress at the tip of microcracks exceeds the safe stress in the environment. The total stress is the sum of internal stresses of the second kind existing at the boundary of parts and additional stresses caused by the pressure of the adsorbed layer. Kinetic calculations based on the mechanism of microcrack growth [26] demonstrate that

(20) $$\sigma_{m}=k_{1}\sigma_{0}/(2+k_{1})$$

where $\sigma_{0}$ is the specific surface energy of a body in a surface-inactive medium; k_1_ is the coefficient determined by the geometry of microcracks and equal to 0.3–2.0. For example, for coals $\sigma_{0}\approx 10^{-2}\frac{J}{m^{2}}$ [6], $\sigma_{m}\approx 13-60\frac{J}{m^{2}}$ [15].

Pertsov A.V. proposed dividing the phenomenon of spontaneous dispersion into three groups of processes: i. True (entropic) spontaneous dispersion, which is not associated with the defective structure of the solid phase and leads to formation of a thermodynamically stable lyophilic colloidal system. ii. Limited swelling, in which the dispersion of the emerging system is uniquely determined by the structure of the initial phase. iii. Quasi-spontaneous dispersion—the spontaneous transformation of solids into dispersed systems in which solid particles are separated by thin layers of dispersed phase [27]. Rebinder and Shchukin previously stated that the presence of dislocations with a density of 10^15^–10^16^ m^−2^ in a solid body, corresponding to a stored energy of 10^7^ J/m^2^, makes the formation of particles with a size of 10^−8^ m thermodynamically favorable, even at an interfacial energy of 10 mJ/m^2^ [28]. During quasi-spontaneous dispersion, some of the system’s free energy associated with its metastability is converted into the surface energy of newly emerging phase interfaces. Similar processes can occur at the interface between two liquids. This can be observed during the “turbulization” of the surface [29] and spontaneous emulsification. There are three main mechanisms of spontaneous emulsification. Interfacial instability is caused by the gradient of interfacial tension during the diffusion of substances across the interface (Marangoni effect), which leads to dispersion and the formation of individual droplets. Dispersion occurs when interfacial tension decreases to almost zero values, which is accompanied by a spontaneous increase in the interfacial surface. Emulsification occurs during the condensation of a new phase in local zones of supersaturation. Dispersion occurs in the first two mechanisms, which involve the mechanical rupture of the interphase surface. The third mechanism involves the formation of a heterogeneous system from a homogeneous one [30]. Pertsov A.V. demonstrated that the work of dispersion (per one particle of radius r_0_ which turns into two particles of radius r) is equal to 4πr^2^ σ_0_, where σ_0_ is the surface tension of a flat surface. This expression also applies to surfactants adsorbed on the interfacial surface, taking into account the dependence of surface tension on the radius of curvature of the surface. The activation energy for the spontaneous fragmentation of a drop due to fluctuations is approximately a quarter of its surface energy: ${∆F}_{a}\approx 0.29(4\pi r_{0}^{2}\sigma )$ [30].

Quasi-spontaneous dispersion upon contact of solid metals with liquid metals manifests itself in the phenomenon of liquid metal embrittlement (LME). The prerequisite for LME is direct contact of liquid and solid metals—wetting. LME manifests itself in the propagation of cracks in solid metal. A crack propagates because of the wetting of grain boundaries because of the capillary effect, providing “negative” pressure and the flow of liquid metal into the crack tip. The tensile stress required to propagate a pre-existing crack can be estimated using the Griffiths equation [31]:

(21) $$S=\sqrt{\frac{A\cdot E{\cdot \sigma }_{sl}}{c}}$$

where c is the length of the crack, A is a constant of order of unity, E is Young’s modulus, and $\sigma_{sl}$ is the free surface energy per unit area of the wetted surface of the crack and is determined by Young’s equation,

(22) $$\cos\left(\theta \right)=\frac{{\sigma_{sl}-\sigma }_{sg}}{\sigma_{lg}}$$

where θ is the contact angle, $\sigma_{sg}$, $\sigma_{lg}$ and $\sigma_{sl}$ are the free surface energy at the steel–gas and liquid metal–gas interfaces, respectively. The “negative” pressure that provides the capillary effect is related to $\sigma_{lg}$ and θ by the Young–Laplace equation:

(23) $$∆P=\frac{2\sigma_{lg}cos(\theta )}{r}$$

where r is the radius of the microchannel.

Currently, there is no clear definition of the concept of LME or a unity of opinions about the mechanism of LME. In particular, it is proposed to group the definitions of LME into scientific schools: (i) elastic-like destruction; (ii) a phenomenon showing strong similarities to stress corrosion cracking or hydrogen embrittlement; (iii) a kinetic process controlled by subcritical crack growth, when, after reaching a threshold value of the stress intensity factor, the crack growth rate sharply increases and does not change further [32]. Various mechanisms of LME have been proposed [33]. The initial mechanism of LME is based on reducing the cohesion of solid atoms through the adsorption of LME onto the surface of a crack. The decrease in surface energy, σ_sl_, caused by adsorption leads to a reduction in the critical stress, S, required for crack propagation, as described by the Griffiths Equation (21) [32]. This is known as the Rehbinder effect [34,35,36]. The Rehbinder effect is immediate and occurs solely as a result of adsorption. The relationship between σ_sl_ and S was initially discovered during experiments involving the treatment of aluminum with Pb, Bi, and Cd melts [37]. Another mechanism of LME, known as the Lynch model, suggests that LME is caused by the emission of dislocations resulting from the adsorption of liquid metal at the crack tip [38,39]. The emission of dislocations is caused by a reduction in the critical shear stress due to adsorption, resulting in localized microplastic deformation. The crack propagates by breaking the shear bands, which is facilitated by adsorption. The Lynch adsorption reduction mechanism is also known as the SJWK model, named after Stoloff–Johnston and Westwood–Kamdar who proposed it independently. The Lynch model accurately predicts the impact of temperature and strain rate on the LME effect. Higher temperatures increase the nucleation and mobility of dislocations, resulting in a greater LME effect and faster crack propagation. Lowering the strain rate at a constant temperature allows for more time for dislocation activation, leading to a stronger LME effect. The third LME mechanism is the Robertson model [40], which is based on the nonequilibrium solubility of solid metal in liquid metal near the crack tip. This model was developed in the works of Glickman [41]. Its main advantage is that it allows for the estimation of the maximum crack growth rate, which can be compared with experimental results. Hadjem-Hamouche et al. conducted a study on the behavior of T91 steel in Pb55Bi melt at temperatures of 160, 250, and 350 °C. They found that the crack rate decreased with increasing temperature [42], which contradicts the Robertson model [40]. Therefore, it can be concluded that the LME effect in this case was caused by the adsorption of liquid metal on the surface of a solid. The answer to the question of whether one mechanism predominates during LME or whether there may be an interaction of several mechanisms has not yet been given.

## 3. Results and Discussion

- **Spontaneous dispersion as a cause of the microstratification of metal melts with different types of phase diagrams**

The phase diagram of Cu–Ni melts is characterized by a continuous series of solid solutions. Results of measurements of kinematic viscosity of melts of the Cu–Ni system with nickel contents of 10, 20, 30, 40, 50, 60, 70, 80 and 90 at % were analyzed within the framework of theory [21], which made it possible to determine the temperatures at which a change in the characteristics of the viscous flow occurs, and hence the “liquidliquid” structural transition [43]. Based on experimental data on the surface tension of Cu–Ni melts [44], the interfacial tension at the boundaries of dispersed particles (Liquid 1) and the melt (Liquid 2) has been evaluated, assuming complete wetting (19). Then, using expression (4) obtained by Reindeer [20], the size of dispersed particles was calculated at a boundary value of surface tension σ_2_, at which spontaneous dispersion becomes thermodynamically favorable (Table 2).

In recent times, researchers have focused on liquid metals at room temperature, particularly Ga–In–Sn alloys with a eutectic phase diagram [45]. Researchers used in situ high-energy X-ray diffraction, thermal expansion, and heat capacity measurements, along with ab initio molecular dynamics simulations, to provide both experimental and theoretical evidence for the existence of a liquid–liquid transition in a eutectic Ga–In–Sn melt at approximately 550 K. This transition is mainly associated with the aggregation of In and Sn atoms [45]. This work is based on experimental and calculated data on the surface tension of Ga–In and Ga–Sn melts [46,47,48,49]. The interfacial surface tension at the boundaries of dispersed particles (Liquid 1) and the melt (Liquid 2) is calculated with the assumption of complete wetting (19). The sizes of dispersed particles (r,nm) were calculated using expression (4) obtained by Rehbinder [20] for the boundary value of surface tension (σ_2_, mJ/m^2^), at which spontaneous dispersion becomes thermodynamically favorable. The results are presented in Table 3.

Alloys with a monotectic phase diagram, such as Fe–Cu and Co–Cu, attract constant attention from researchers. Measurements of the kinematic viscosity of melts in the Fe–Cu system have allowed for the determination of the temperatures at which a change in the characteristics of the viscous flow occurs, indicating a “liquid–liquid” structural transition [50]. Measurements of electrical resistivity of liquid Cu20Co80 and Cu60Co40 alloys have revealed the possibility of a liquid–liquid transition [51]. This work uses experimental data on the surface tension of Fe–Cu [44] and Co–Cu [52,53,54] melts to determine the interfacial surface tension at the boundaries of dispersed particles (Liquid 1) and the melt (Liquid 2) with the assumption of complete wetting (19). The sizes of dispersed particles (r, nm) were calculated using expression (4) obtained by Rehbinder [20] for the boundary value of surface tension (σ_2_, J/m^2^), at which spontaneous dispersion becomes thermodynamically favorable (Table 4).

In the context of discussing the results (Table 1) obtained, a comment is necessary because many properties of substances in the ultradisperse state significantly depend on the particle size (d). For example, in the range d < 1 nm, an increase in the strength of crystals is observed with a decrease in their diameter d. In order, this “threshold” corresponds to the average distance between dislocations in crystals. B.D. Summ notes that for nanosystems it is necessary to consider the dependence of surface tension on particle size. Typically, Tolman’s formula is used to analyze the scale (size) dependence of surface tension [55]:

(24) $$\sigma_{r}\cdot \left(1+\frac{2\delta }{r}\right)=\sigma_{0}$$

where σ_r_ is the surface tension corresponding to the curvature of radius r, σ_0_ is the surface tension for a flat surface, and δ is the thickness of the surface layer, characterized (according to Gibbs) by excess thermodynamic functions. According to a number of estimates, δ≈1 nm. Consequently, a significant change (increase) in surface tension is observed for nanoparticles and gas bubbles [56]. However, it has been experimentally established that a significant change in surface tension is observed if the radius of the drop changes from 1 to 0.1 nm; for example, from 891.23 to 659.9 mJ/m^2^ (Ag) or from 1721.94 to 1329.31 mJ/m^2^ (Ni) [57].

Thus, the phenomenon of spontaneous dispersion can be considered as the cause of the microstratification of metal melts. Microseparation is characterized by the presence of dispersed particles enriched in one of the components suspended in a medium of a different composition with an interphase surface. Microseparation corresponds to a violation of long-range order in the arrangement of liquid metal (LM) atoms and a range of colloidal particle sizes greater than 2 nm. Microseparation occurs due to spontaneous dispersion upon contact of liquid and solid metal or the mixing of two liquid metals. The phenomenon of spontaneous dispersion can be correctly divided into two phenomena that are different in nature: true spontaneous dispersion of particles to colloidal sizes with the formation of thermodynamically equilibrium lyophilic colloidal systems and quasi-spontaneous dispersion, leading to the formation of relatively stable systems prone to collective recrystallization and the formation of large dispersions. The possibility of spontaneous dispersion was assessed using three different criteria: Volmer’s criterion, Rehbinder’s criterion and the diffusion rate criterion. Quasi-spontaneous dispersion upon contact of solid metals with liquid metals manifests itself in the phenomenon of liquid metal embrittlement (LME). A prerequisite for LMC is the direct contact of liquid and solid metals—wetting. LME manifests itself in the propagation of cracks in solid metal. The crack propagates as a result of the wetting of the grain boundaries as a result of the capillary effect, providing “negative” pressure and the flow of liquid metal to the crack tip. There are currently three known LMO mechanisms: the Rehbinder effect, the Lynch model and the Robertson model. The conditions of spontaneous dispersion when mixing melts as the cause of microstratification were analyzed. The Rebinder criterion was used to assess the possibility of spontaneous dispersion in liquid alloys with different types of phase diagrams. The interfacial tension between dispersed particles (liquid 1) and the melt (liquid 2) was calculated assuming complete wetting. This allowed us to determine the maximum sizes of the dispersed particles. For the (Cu10Ni90-506 Cu20Ni80) dispersion with a particle size of 2 nm, there are violations of the medium-range order (MRO) in a range of 0.5–2 nm. The (Ga50In50-Ga50Sn50) dispersion has a particle size of 5.6 nm. The (Cu80Fe20-Cu60Fe40) dispersion has a particle size of 4.8 nm and corresponds to a disruption of long-range order (LRO) with a range greater than 2 nm.

## 4. Conclusions

1. The diffusion rate criterion for true (entropy) spontaneous dispersion was obtained, which coincides with the Volmer criterion and the Rehbinder criterion up to a factor. The criterion diffusion rate criterion was obtained on the basis of the theory of high-speed processes [21], which describes how diffusing atoms overcome the interphase boundary.

2. The possibility of spontaneous dispersion was assessed using three different criteria: Volmer’s criterion, Rehbinder’s criterion and the diffusion rate criterion. The diffusion rate criterion was used to assess the value of interfacial tension in order to predict the maximum possible sizes of microinhomogeneities in eutectic Al–Si, Al–Ge and Al–Sn melts. It was established that in Al–Sn melts, the size of microinhomogeneities has a colloidal scale (4 nm), and in liquid Al–Si and Al–Ge eutectics, it is on an atomic scale (0.1 nm).

3. Using the Rehbinder criterion, the conditions of spontaneous dispersion when mixing liquid alloys with different types of phase diagrams were analyzed. The maximum sizes of dispersed particles were obtained for: dispersion (Cu10Ni90-Cu20Ni80)—2 nm; dispersion (Ga50In50-Ga50Sn50)—5.6 nm; and dispersion (Cu80Fe20-Cu60Fe40)—4.8 nm.

## Figures and Tables

**Table 1:** Interfacial tension at the “dispersed particle–melt” boundary at T = 970 K.

Melt	d (Heating)/ d (Cooling)	d (Heating), nm	σ, mJ/m^2^	σ (Heating)/ σ (Cooling)
Al97Sn3	2.1	0.9	8.62	4.4
Al95Sn5	2.3	4.0	0.42	5.3
Al91Sn9	5.2	2.0	1.68	27.0
Al81Sn19	11.1	2.0	1.68	123.2
Al52Sn48	3.3	3.0	0.74	10.9
Al33Sn67	1.5	0.7	2.98	2.2
Al95Si5	1.0	0.1	669.30	1.0
Al80Sn20	1.0	0.2	167.33	1.0
Al90Ge10	1.0	0.3	74.36	1.0
Al60Sn40	6.8	1.4	3.41	26.2

**Table 2:** Interfacial tension σ_2_ (J/m^2^) at the boundaries of dispersed particles (Liquid 1) of radius r (nm) and melt (Liquid 2), at which spontaneous dispersion becomes thermodynamically favorable. Spontaneous dispersion as a cause of the microstratification of the melts in a Cu–Ni system. The phase diagram of Cu–Ni melts is characterized by a continuous series of solid solutions.

Liquid 1	Liquid 2	The Interfacial Tension (J/m^2^)	$\sigma_{2}$ (J/m^2^)	r (nm)
Cu10Ni90	Cu90Ni10	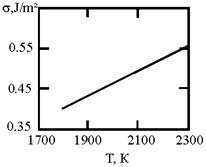	0.4	1
Cu10Ni90	Cu70Ni30	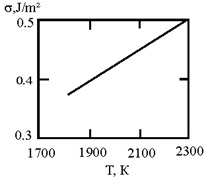	0.4	1
Cu10Ni90	Cu30Ni70	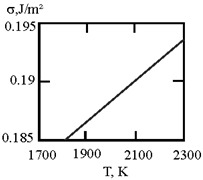	0.1	2
Cu10Ni90	Cu20Ni80	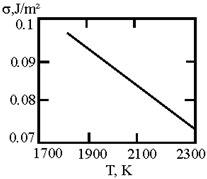	0.08	2
Cu70Ni30	Cu30Ni70	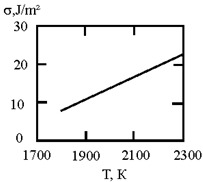	20	0.2

**Table 3:** Interfacial tension σ_2_ (J/m^2^) at the boundaries of dispersed particles (Liquid 1) of radius r (nm) and melt (Liquid 2), at which spontaneous dispersion becomes thermodynamically favorable. Spontaneous dispersion as a cause of microstratification of the eutectic Ga–In–Sn melt. The Ga–In–Sn alloys have a eutectic phase diagram.

Liquid 1	Liquid 2	The Interfacial Tension (J/m^2^)	$\sigma_{2}$ (J/m^2^)	r (nm)
Ga90In10	Ga90Sn10	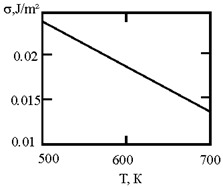	0.015	3.1
Ga80In20	Ga90Sn10	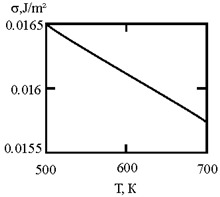	0.016	3.1
Ga80In20	Ga90Sn10	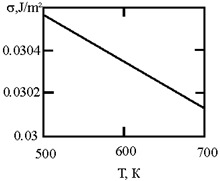	0.0302	2.2
Ga90In10	Ga70Sn30	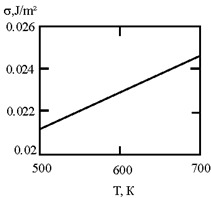	0.022	2.6
Ga50In50	Ga50Sn50	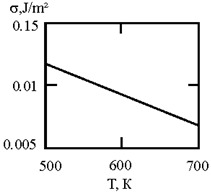	0.005	5.4

**Table 4:** Interfacial tension σ_2_ (J/m^2^) at the boundaries of dispersed particles (Liquid 1) of radius r (nm) and melt (Liquid 2), at which spontaneous dispersion becomes thermodynamically favorable. Spontaneous dispersion as a cause of the microstratification of the melt Cu–Fe and Cu–Co systems. Phase diagrams of Cu–Co and Fe–Cu showing the metastable miscibility gap.

Liquid 1	Liquid 2	The Interfacial Tension (J/m^2^)	$\sigma_{2}$ (J/m^2^)	r (nm)
Cu80Fe20	Cu20Fe80	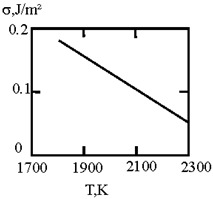	0.1	2.2
Cu80Fe20	Cu60Fe40	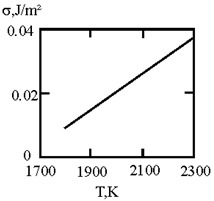	0.02	4.8
Cu80Fe20	Cu40Fe60	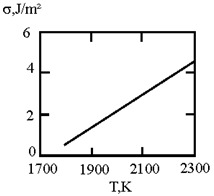	2	0.5
Co85Cu15	Co10Cu90	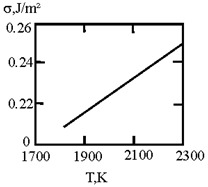	0.22	1.5

## Data Availability

The raw data supporting the conclusions of this article will be made available by the authors on request.

## References

[1] Chikova O.A. (2020). Structural transitions in complexly alloyed melts. Izv. Ferr. Metall..

[2] Tsepelev V.S., Chikova O.A., Shmakova K.Y. (2022). Model of temperature-induced liquid–liquid transition in metallic melts. Metall. Mater. Trans. B.

[3] Popel P.S. (2005). Metastable microheterogeneity of melts in systems with eutectic and monotectic and its influence on the structure of the alloy after solidification. Melts.

[4] Calvo-Dahlborg M., Popel P.S., Kramer M.J., Besser M., Morris J.R., Dahlborg U. (2013). Superheat-dependent microstructure of molten Al–Si alloys of different compositions studied by small angle neutron scattering. J. Alloys Compd..

[5] Klyachko Y.A. (1935). The Experience of Colloidal Chemical Research of Metals.

[6] Vertman A.A. (1967). Microheterogenicity of metal melts and the problem of regulating the properties of castings. Phys. Chem. Mater. Process..

[7] Zalkin V.M. (2005). On the microheterogeneous structure of eutectic alloys (solutions) in the liquid state. J. Phys. Chem..

[8] Frenkel Y.I. (1948). Statistical Physics.

[9] Morokhov D.I., Trusov L.I., Lapovok V.N. (1984). Physical Phenomena in Ultrafine Media.

[10] Chikova O.A., Tsepelev V.S., Moskovskikh O.P. (2017). Estimation of parameters of the microheterogenic structure of metal alloys from the results of a viscometric experiment based on representations of the theory of absolute reaction rates. J. Phys. Chem..

[11] Kaptay G. (2017). On the Negative Surface Tension of Solutions and on Spontaneous Emulsification. Langmuir.

[12] Sinitsin N.I., Chikova O.A. (2022). Thermodynamic stability of microheterogenous states in Fe—Mn—C melts. Izv. Ferr. Metall..

[13] Chikova O.A., Sinitsin N.I., V’yukhin V.V. (2022). Surface Tension of Fe–Mn–C Melts. Rus. J. Phys. Chem. A.

[14] Chikova O.A. (2008). Spontaneous dispersion in alloy formation processes as a cause of micro-stratification of metal melts. Melts.

[15] Makeiev S., Andreiev S., Ryzhov H. (2019). The study of the process of physic-chemical destruction of coal by the method of physical modeling. In proceeding International Conference Essays of Mining Science and Practice. E3S Web Conf..

[16] Shchukin E.D. (1996). The influence of surface-active media on the mechanical properties of materials. Adv. Coll. Interface Sci..

[17] Shchukin E.D., Pertsov A.V., Tadros T.F. (2011). Thermodynamic Criterion of Spontaneous Dispersion. Colloid Stability: The Role of Surface Forces.

[18] Shchukin E.D. (2005). Conditions of spontaneous dispersion and formation of thermodynamically stable colloid systems. J. Dispers. Sci. Technol..

[19] Volmer M. (1957). Die kolloidale Natur von Fluessigkeitsgemischen in der Umgebung des kritischen Zustandes. Z. Phys. Chem..

[20] Rebinder P.A. (1979). Surface phenomena in disperse systems. Physico-Chemical Mechanics: Selected Works.

[21] Glasstone S., Laidler K.J., Eyring H. (1941). The Theory of Rate Processes: The Kinetics of Chemical Reactions, Viscosity, Diffusion and Electrochemical Phenomena.

[22] Summ B.D., Il’ichev E.Y. (1996). Correlation between Surface Tension of Pure Liquids and Their Heats of Melting. Russ. J. Phys. Chem. A.

[23] Summ B.D. (1995). The relationship between surface tension and melting heat of alkali metals. Inorg. Mater..

[24] Summ B.D. (2005). Phase transitions in the surface layer and the surface tension of liquids. Russ. J. Phys. Chem. A.

[25] Kaptay G. (2015). Partial Surface Tension of Components of a Solution. Langmuir.

[26] Brtenev G.M., Yudina I.V., Rebinder P.A. (1958). The theory of spontaneous dispersion of solids. Colloid J..

[27] Pertsov A.V. (2005). Quasi-spontaneous dispersion of solids. Colloid J..

[28] Shchukin E.D., Rebinder P.A. (1958). Formation of new surfaces during deformation and destruction of solids in a surface-active medium. Colloid J..

[29] Sanfeld A., Lin M., Bios A., Panaiotov I., Baret J.F. (1984). Mechanical and electrochemical effects on surface convection and on emulsification new criteria. Adv. Colloid. Interface Sci..

[30] Koroleva M.Y., Yurtov E.V. (2012). Nanoemulsions: The properties, methods of preparation and promising applications. Russ. Chem. Rev..

[31] Griffith A.A. (1921). The Phenomena of Rupture and Flow in Solids. Phil. Trans. R. Soc. Lond. A.

[32] Gorse D., Auger T., Vogt J.B., Serre I., Weisenburger A., Gessi A., Agostini P., Fazio C., Hojna A., Di Gabriele F. (2011). Influence of liquid lead and lead–bismuth eutectic on tensile, fatigue and creep properties of ferritic/martensitic and austenitic steels for transmutation systems. J. Nucl. Mater..

[33] Fernandes P.J.L., Jones D.R.H. (1997). Mechanisms of liquid metal induced embrittlement. Int. Mater. Rev..

[34] Rehbinder P.A., Shchukin E.D. (1972). Surface phenomena in solids during deformation and fracture processes. Progress Surf. Sci..

[35] Shchukin E.D., Savenko V.I., Malkin A.I. (2013). The effect of a surface-active medium on the mechanical stability and damageability of a solid surface. Review. Protection Met. Phys. Chem. Surf..

[36] Malkin A.I. (2012). Regularities and mechanisms of the Rehbinder’s effect. Colloid J..

[37] Roth M.C., Weatherly G.C., Miller W.A. (1980). The temperature dependence of the mechanical properties of aluminum alloys containing low-melting-point inclusions. Acta Metall..

[38] Lynch S.P. (1984). Metal induced embrittlement of ductile materials and dislocation emission from crack tips. Scripta Metall..

[39] Lynch S.P. (1989). Metallographic contributions to understanding mechanisms of environmentally assisted cracking. Metallography.

[40] Robertson W.M. (1966). Propagation of a Crack Filled with Liquid Metal. Trans. Met. Soc. AIME.

[41] Glickman E. (2007). On the Role of Stress, Strain and Diffusion in Dissolution—Condensation Mechanism of Liquid Metal Embrittlement. Defect Diffus. Forum..

[42] Hadjem-Hamouche Z., Auger T., Guillot I. (2009). Temperature effect in the maximum propagation rate of a liquid metal filled crack: The T91 martensitic steel/Lead–Bismuth Eutectic system. Corros. Sci..

[43] Chikova O.A., Tkachuk G.A., V’yukhin V.V. (2019). Viscosity of Cu–Ni melts. Russ. J. Phys. Chem. A.

[44] Brillo J., Egry I. (2005). Surface tension of nickel, copper, iron and their binary alloys. J. Mat. Sci..

[45] Wang X., Yu Q., Wang X., Dai Z., Cao Q., Ren Y., Zhang D., Jiang J.Z. (2021). Temperature-Induced Structural Changes in the Liquid GaInSn Eutectic Alloy. J. Phys. Chem. C.

[46] Dadashev RKh Kutuev R.A., Elimkhanov D.Z., Bichueva Z.I. (2007). Surface tension of Indium-Tin-Gallium melts. Rus. J. Phys. Chem. A.

[47] Alchagirov B.B., Dyshekova F.F., Dadashev R.K., Elimkhanov D.Z. (2014). The surface tension of indium: Methods and results of investigations. High Temp..

[48] Alchagirov B.B., Mozgovoi A.G. (2005). The surface tension of molten Gallium at high temperatures. High Temp..

[49] Yan L., Zheng S., Ding G., Xu G., Qiao Z. (2007). Surface tension calculation of the Sn–Ga–In ternary alloy. Comp. Coup. Phase Diagr. Thermochem..

[50] Chikova O.A., Tsepelev V.S., V’Yukhin V.V., Konstantinov A.N. (2014). Viscosity of Fe-Cu melts. Inorg. Mater..

[51] Guo F., Lu T., Qin J., Zheng H., Tian X. (2012). Abnormal resistivity behavior of Cu–Ni and Cu–Co alloys in undercooled liquid state. Phys. B Condens. Matter.

[52] Guo F., Wang W., Wang Q., Yuan J., Li L., Jiang H., Zhou Z., Tian X. Phase Separation and Electrical Resistivity of Liquid Cu-Co Alloys. Proceedings of the International Symposium on Material, Energy and Environment Engineering (ISM3E 2015).

[53] Eichel R.A., Egry I. (1999). Surface tension and surface segregation of liquid cobalt-iron and cobalt-copper alloys. Int. J. Mat. Res..

[54] Egry I., Ratke L., Kolbe M., Chatain D., Curiotto S., Battezzati L., Johnson E., Pryds N. (2010). Interfacial properties of immiscible Co–Cu alloys. J. Mater Sci..

[55] Tolman R.C. (1949). The effect of droplet size on surface tension. J. Chem. Phys..

[56] Summ B.D., Ivanova N.I. (2000). The use of objects and methods of colloid chemistry in nanochemistry. Russ. Chem. Rev..

[57] Shebzukhova M.A., Shebzukhov Z.A., Shebzukhov A.A. (2010). The Tolman parameter, self-absorption, and surface tension on flat and curved surfaces of liquid metals. Bull. Russ. Acad. Sci. Phys..

